# Controlled synthesis of high-density metal atom interface defects for acid water oxidation

**DOI:** 10.1093/nsr/nwaf177

**Published:** 2025-05-02

**Authors:** Xinyu Ping, Yurui Xue, Han Wu, Siao Chen, Siyi Chen, Yang Gao, Yuliang Li

**Affiliations:** CAS Key Laboratory of Organic Solids, Institute of Chemistry, Chinese Academy of Sciences, Beijing 100190, China; CAS Key Laboratory of Organic Solids, Institute of Chemistry, Chinese Academy of Sciences, Beijing 100190, China; State Key Laboratory of Supramolecular Structure and Materials, College of Chemistry, Jilin University, Changchun 130012, China; CAS Key Laboratory of Organic Solids, Institute of Chemistry, Chinese Academy of Sciences, Beijing 100190, China; School of Chemical Sciences, University of Chinese Academy of Sciences, Beijing 100190, China; CAS Key Laboratory of Organic Solids, Institute of Chemistry, Chinese Academy of Sciences, Beijing 100190, China; School of Chemical Sciences, University of Chinese Academy of Sciences, Beijing 100190, China; CAS Key Laboratory of Organic Solids, Institute of Chemistry, Chinese Academy of Sciences, Beijing 100190, China; School of Chemical Sciences, University of Chinese Academy of Sciences, Beijing 100190, China; CAS Key Laboratory of Organic Solids, Institute of Chemistry, Chinese Academy of Sciences, Beijing 100190, China; CAS Key Laboratory of Organic Solids, Institute of Chemistry, Chinese Academy of Sciences, Beijing 100190, China; School of Chemical Sciences, University of Chinese Academy of Sciences, Beijing 100190, China

**Keywords:** graphdiyne, proton exchange membrane water electrolyser, acid oxygen evolution reaction, metal atom interface defects

## Abstract

Controlled selective growth of an interface with high-density active sites is the key to constructing efficient and long-term stable proton exchange membrane water electrolysis, which can effectively promote an acidic oxygen evolution reaction. Herein, a 2D all-carbon graphdiyne (GDY) is used as an ideal support to grow a new generated interface of RuO*_x_*/GDY and finally to achieve the directed and controlled production of high-density Ru atom defects. The metal atom defect-rich interface not only afforded high intrinsic activity by facilitating the adsorption/desorption ability of reaction intermediates, but also enhanced structural stability by forming interfacial chemical bonds. RuO*_x_*/GDY shows a small overpotential of 157 mV at 10 mA cm^−2^ and 100 h of stability in acidic electrolyte. The proton exchange membrane water electrolyser when using RuO*_x_*/GDY as the anodic catalyst only requires 1.47 V to achieve 1000 mA cm^−2^ and the estimated cost of hydrogen production is US $0.78 kg^−1^ H_2_.

## Teaser

This work reported the controlled synthesis of high-density Ru defects under the assistance of graphdiyne (GDY). The proton exchange membrane water electrolyzer using RuOx/GDY as the anodic catalyst exhibits a record-low potential of 1.47 V at 1000 mA cm-2 for acid water oxidation.

## INTRODUCTION

Water electrolysis in a proton exchange membrane water electrolyser (PEMWE) effectively addresses the shortcomings of alkaline and neutral water electrolysis, such as slow current response, more side reactions and high ohmic resistance, making it the most mainstream way for hydrogen production [1–3]. Achieving efficient and sustainable water splitting in PEMWE is of great importance to the hydrogen production industry, which has been severely hampered by the inefficiency and instability of the traditional acidic oxygen evolution reaction (OER) catalysts [4,5]. During past decades, various strategies have been proposed to synthesize high-performance electrocatalysts. Among them, defect engineering has emerged as a promising strategy to enhance the conductivity of catalysts and optimize the adsorption energies of intermediates, resulting in an improvement in overall catalytic performances [6–8]. However, these traditional strategies have been largely limited in practical applications [9,10]. For example, Ge's group [11] confirmed that doping RuO₂ with higher electronegative elements increases the proportion of the lattice oxygen mechanism (LOM) pathway during OER, leading to poor stability. Similarly, Zhao *et al*. [12] showed that excessive defects can irreversibly accelerate catalyst structural collapse by promoting the LOM pathway. Unlike non-metallic atom defects, metal atom defects offer additional advantages, including higher structural stability, a wider energy-band center control range and better catalytic activity [13]. However, studies on high-performance catalysis systems by the fabrication of metal atom defects have been rarely reported. Constructing a new interface with high-density metal atom defects as the active site and achieving high selectivity and high yield of the interface is our original intention to explore the difficult catalytic reaction and it is also the only way to develop the current transformative catalytic system.

Ruthenium-based materials have been widely used as OER electrodes in PEMWEs, but their activity and stability are far from the practical needs [14]. In the past few decades, the high formation energy has meant that there is no efficient way to fabricate metal atom defects. Fortunately, the discovery of the graphdiyne (GDY) material—a novel carbon allotrope with a 2D highly conjugated structure composed of a benzene ring and an acetylene linker in which the super-large conjugated structure of GDY and its natural hollow structure play a powerful supporting role for metal atoms and can control multiple effects at the surface and interface—made the impossible become possible [15–17]. GDY has changed the possibility that traditional carbon materials (sp^2^ hybridization) cannot synthesize catalysts with metal atom defects [18,19]. With unique structural advantages (such as a rich three-bond conjugated structure and efficient transport capacity, excellent electrochemical stability and uneven surface charge distribution, etc.), the distinctive characteristics of GDY itself provide a rare opportunity for the development of efficient catalytic systems [20–24]. The presence of *sp*- and *sp*^2^-cohybridized carbon in GDY results in uneven charge distribution and high-density distribution of abundant acetylene bonds on its surface, which can be easily customized to regulate the electronic structure of the metal atoms, control the production of the required metal atom defects and achieve the purpose of essentially changing the catalytic activity [25–27]. Also, the electron-rich *sp*-C can not only anchor metal atoms, but also act as an electron reservoir, which can prevent the excessive oxidation and dissolution of metal atoms, significantly improving the stability of the catalysts [28–31]. Combined with previous work, we accurately propose to anchor RuO_2_ on the surface of GDY through the reduction of RuO_2_, create high-density Ru atom defects on the interface and finally achieve excellent acidic OER performance.

In such challenging material-preparation work, our creative idea is to induce the controllable selective growth of high-density Ru atom defects on interfaces of RuO*_x_*/GDY through the strong reducibility of GDY and interaction with oxides. The experimental results show that our prediction is accurate and the continuous incomplete charge transfer between metal atoms and GDY achieves dynamic equilibrium in the system, thus greatly improving the catalytic activity and stability. Density functional theory (DFT) calculations demonstrate that RuO*_x_*/GDY with Ru atom defects exhibits a lower Ru 4*d*- and O 2*p*-band center as compared with that of pure RuO*_x_*, resulting in the optimal adsorption/desorption of reaction intermediates and efficient suppression of the LOM pathway during the acidic OER process. As expected, the as-synthesized RuO*_x_*/GDY catalyst exhibits extremely low overpotentials of 157 and 201 mV at 10 and 100 mA cm^−2^, respectively, as well as excellent stability. When it was used as the anode catalyst of the PEMWE set-up, the electrolyser reached large current densities of 1000 and 2000 mA cm^−2^ at very small applied voltages of 1.47 and 1.52 V, respectively, with an energy consumption of 3.51 kWh Nm^−3^ H_2_ at 1000 mA cm^−2^ and the much lower cost of US$0.78 per kg^−1^ H_2_ than the general international target (US$2.0 per kg^−1^ of H_2_).

## RESULTS AND DISCUSSION

### Synthesis and structural characterization of the samples

**Figure 1.**
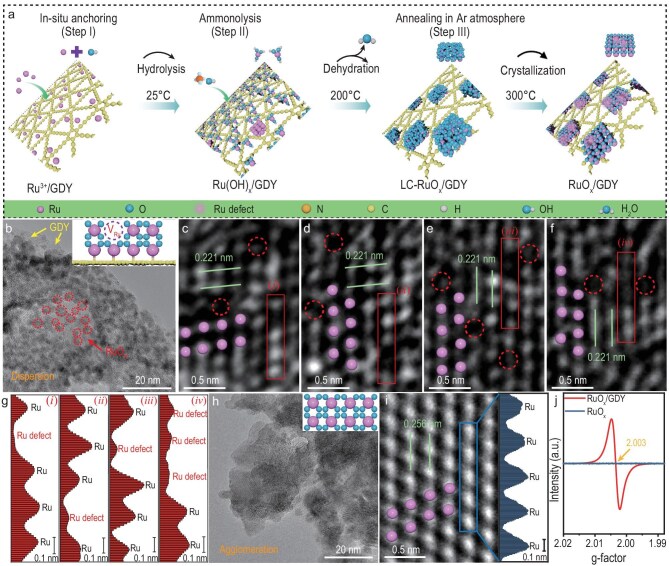
Synthesis and morphology characterizations of RuO*_x_*/GDY. (a) Illustration of the synthesis of RuO*_x_*/GDY and structural evolution process of RuO*_x_*/GDY with increasing temperature; LC-RuO*_x_*/GDY: low-crystallized RuO*_x_*/GDY. (b) TEM and (c–f) HRTEM images of RuO*_x_*/GDY. (g) The corresponding intensity profile along the solid rectangles in (c–f). (h) TEM and (i) HRTEM images of pure RuO*_x_* and the corresponding intensity profile along the solid rectangle. (j) Electron paramagnetic resonance spectra of RuO*_x_*/GDY and RuO*_x_*.

The RuO*_x_*/GDY interface structure with high-density metal atom defects was synthesized through a three-step method (Fig. 1a), which involves the initial anchoring of Ru atoms onto GDY surfaces (Step I), followed by hydrolysis treatment to yield Ru(OH)*_x_* (Step II) and finally a decomposition process to form RuO*_x_* (Step III). The morphological and structural evolution of the as-synthesized samples were analysed by using scanning electron microscopy (SEM), high-resolution transmission electron microscopy (HRTEM), X-ray diffraction and thermogravimetric analysis (TGA). As shown in Fig. S1, RuO*_x_*/GDY samples exhibit a rougher surface than that of GDY, which indicates the formation of Ru-containing species on the GDY surface. HRTEM images (Figs S2 and S3) show the presence of small particles with lattice spacing of 0.20 nm corresponding to Ru metal on the GDY surface after hydrolysis treatment at 25°C. As the annealing temperature increased, the Ru(OH)*_x_* species dehydrated and new RuO*_x_* with different crystallinity was obtained at 200°C and 300°C, respectively. Interestingly, the highly dispersed small RuO*_x_* nanoparticles finally grew into a Ru metal nanoplate with the assistance of carbothermic reduction at 400°C (Fig. S3). This inference was also confirmed by the TGA curve (Fig. S4), meaning the complete conversion of Ru(OH)*_x_*/GDY to RuO*_x_*/GDY at 300°C.

Transmission electron microscopy (TEM) images of RuO*_x_*/GDY show that RuO*_x_* particles are uniformly distributed on the surface of the GDY nanosheets, with an average particle size of 2.93 ± 0.02 nm (Fig. 1b and Fig. S5). The lattice spacing observed is 0.22 nm, consistent with the (200) plane of RuO_2_ (Fig. 1c–f). Notably, high-density Ru atom defects are observed in RuO*_x_*/GDY as confirmed by the atom line profiles analysis results (Fig. 1g). Atomic force microscopy measurements determined that the thickness of the RuO*_x_* layer in RuO*_x_*/GDY is 0.44 nm (Fig. S6). Energy-dispersive spectroscopy (EDS) results confirmed the distribution of the C, O and Ru elements throughout the entire RuO*_x_*/GDY (Fig. S7). For comparison, RuO*_x_* was prepared on carbon cloth (CC) without GDY by using the same synthetic method. SEM and TEM images showed that RuO*_x_* possesses larger and more irregular grain size, along with obviously disordered and agglomerated spatial structures (Fig. 1h and Fig. S8). Notably, RuO*_x_* without GDY shows no obvious structural distortion and metal atom defects (Fig. 1i and Fig. S9). Electron paramagnetic resonance further verified the presence of unpaired electrons in RuO*_x_*/GDY due to Ru atom defects but not in RuO*_x_* (Fig. 1j and Fig. S10) [32]. The above results implied that GDY plays a critical point in dispersing particles and creating high-density Ru atom defects, which enhance catalytic performance.

**Figure 2.**
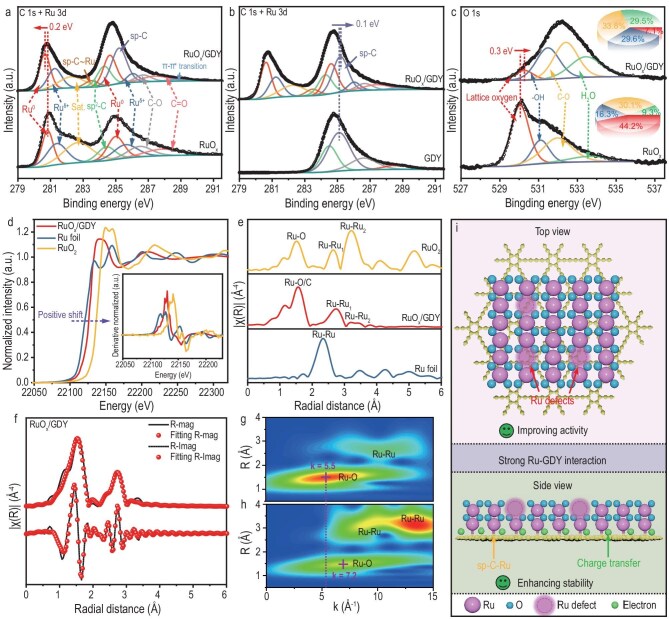
Electronic structure characterizations. (a–c) C 1*s*, Ru 3*d* and O 1*s* XPS spectra of RuO*_x_*/GDY, RuO*_x_* and GDY; (d) X-ray absorption near-edge structure (XANES) and (e) Fourier transform extended X-ray absorption fine structure (FT-EXAFS) spectra of RuO*_x_*/GDY, RuO_2_ and Ru foil (inset: the first derivative of XANES spectra); (f) FT-EXAFS fitting in R-space for RuO*_x_*/GDY; WT-EXAFS spectra of (g) RuO*_x_*/GDY and (h) RuO_2_. (i) Schematic illustration of the strong interaction between GDY and Ru.

Compared with pure RuO*_x_*, the C 1*s* and Ru 3*d* X-ray photoelectron spectroscopy (XPS) spectra of RuO*_x_*/GDY (Fig. 2a) show two additional peaks at 283.5 eV (*sp*-C–Ru) and 289.0 eV (π–π* transition), which reveals the chemical interaction between RuO*_x_* and GDY [33–35]. The Ru 3*d* XPS spectra contain a mixed phase structure of Ru^0^ (representing low-valence Ru species) and Ru^δ+^ (representing high-valence Ru species). Concretely, for RuO*_x_*/GDY, the ratio of Ru^0^ to Ru^δ+^ is 0.89, which is higher than that of RuO*_x_* (0.70), demonstrating that Ru in RuO*_x_*/GDY is in a lower-valence state than RuO*_x_*. This phenomenon has been proved by the position of the Ru^0^ peak. The mixed valence states can induce different electronic defect states on the surface of the catalyst. Especially in the low oxidation state species, it promotes nucleophilic attack and O–O bond formation, which helps in the improvement of catalytic activity [36]. In addition, the low Ru oxidation state improves the stability of the catalyst by preventing the overoxidation and subsequent dissolution of high-valence Ru species (RuO_4_^2−^ species) [37,38]. Furthermore, the binding energy of *sp*-C and lattice oxygen (M–O bond) in RuO*_x_*/GDY also shifts positively by 0.1 and 0.3 eV relative to the pure GDY and RuO*_x_*, respectively (Fig. 2b and c), suggesting that some electrons were transferred from C and O to Ru [39,40]. The presence of a large number of oxygen-containing species (M–OH, C–O and absorbed H_2_O) on the surface of RuO*_x_*/GDY suggests that it has a higher oxygen affinity (Fig. 2c). Raman spectroscopy of RuO*_x_*/GDY exhibits three characteristic peaks for the D band (1398 cm^−1^), G band (1592 cm^−1^) and triple bonds (2161 cm^−1^) (Fig. S11). The higher D/G band intensity ratio for RuO*_x_*/GDY (0.638) indicates an increased density of defects in RuO*_x_*/GDY. Interestingly, the peak for acetylenic bonds in pure GDY (2175 cm^−1^) negatively shifts to 2161 cm^−1^ in RuO*_x_*/GDY, confirming the formation of chemical bonds between RuO*_x_* and GDY. The X-ray absorption near-edge structure spectra reveal that the absorption energy of RuO*_x_*/GDY lies between that of Ru foil and RuO_2_ (Fig. 2d), suggesting a low oxidation state of Ru in RuO*_x_*/GDY, agreeing with the XPS results. The Fourier transform extended X-ray absorption fine structure (FT-EXAFS) spectrum of RuO*_x_*/GDY exhibits two prominent peaks at 1.57 and 2.74 Å, and a weak peak at 3.20 Å, which are attributed to the Ru–O/C, Ru–Ru_1_ and Ru–Ru_2_ scattering paths, respectively (Fig. 2e). To precisely obtain the structural parameters of RuO*_x_*/GDY and RuO_2_, the FT-EXAFS spectra were reasonably fitted (Fig. 2f, Fig. S12 and Table S1). The fitting results revealed that the bond lengths for Ru–O/C, Ru–Ru_1_ and Ru–Ru_2_ in RuO*_x_*/GDY and RuO_2_ are 2.01, 3.11, 3.37, 1.97, 3.08 and 3.55 Å, respectively, meaning that GDY distorts the crystal structure of RuO*_x_*. The fitting results of the coordination number show that each Ru atom in RuO*_x_*/GDY is coordinated with ∼4.07 O/C atoms and ∼5.32 Ru atoms (Table S1)—lower than those of 6 for O atoms and 10 for Ru atoms in RuO_2_—which indicates that Ru atom defects are prevalent in RuO*_x_*/GDY. Wavelet transform analysis with k-space resolution further verifies the presence of the *sp*-C–Ru bonds due to the lower *k* values in the first shell of RuO*_x_*/GDY (Fig. 2g) as compared with RuO_2_ (Fig. 2h). These results suggest that the *sp*-C–Ru interaction between GDY and RuO*_x_* alters the electronic state of Ru through the reduction of RuO*_x_* and induces the formation of Ru atom defects. It is widely accepted that metal atom defects with multifarious electron and orbital distributions can optimize the adsorption energy of oxygen-containing intermediates to enhance the OER performance [6,41,42]. Meanwhile, the formation of *sp*-C–Ru bonds mitigates the dissolution of Ru and ensures the high stability of the electrocatalysts (Fig. 2i).

### Acidic OER performances of RuO*_x_*/GDY-based PEMWEs

**Figure 3.**
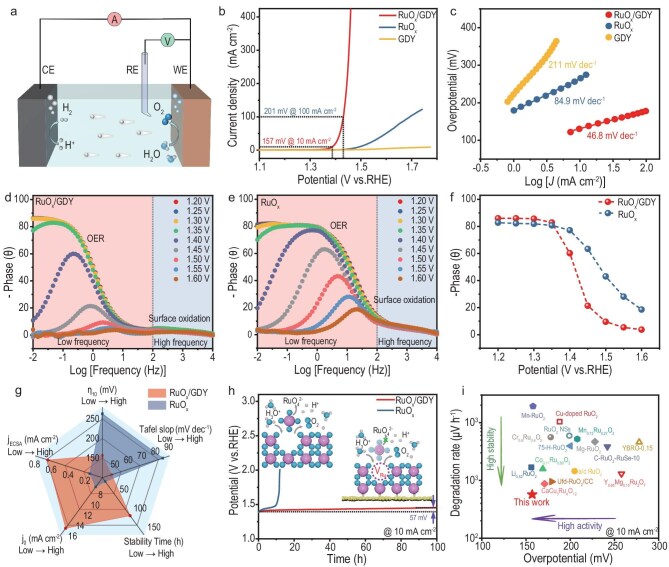
Catalytic activity for OER in 0.5 M H_2_SO_4_. (a) Structural diagram of the three-electrode system for electrochemical water splitting in acidic solution; (b) Linear sweep voltammetry curves for OER of RuO*_x_*/GDY, RuO*_x_* and GDY; (c) Tafel slopes derived from (b); (d and e) Bode phase plots of RuO*_x_*/GDY and RuO*_x_*. (f) Summarized phase peak angles of RuO*_x_*/GDY and RuO*_x_* within the voltage range of 1.20–1.60 V. (g) Comparison of the OER performance of RuO*_x_*/GDY and RuO*_x_*. (h) Chronopotentiometry performance of RuO*_x_*/GDY and RuO*_x_* at 10 mA cm^−2^. (i) Comparison of the overpotential and degradation rate of RuO*_x_*/GDY at 10 mA cm^−2^ with recently reported Ru-based oxide catalysts.

The OER performances of all samples in the 0.5 M H_2_SO_4_ solution were studied by using a three-electrode set-up (Fig. 3a). As shown in Fig. 3b, pure GDY exhibits negligible OER performance. Meanwhile, RuO*_x_* shows relatively poor activity. However, when RuO*_x_* grows on GDY, the RuO*_x_*/GDY catalyst manifests superior activity with low overpotentials of 157 and 201 mV at 10 and 100 mA cm^−2^, respectively. Additionally, RuO*_x_*/GDY exhibits the smallest Tafel slop of 46.8 mV dec^−1^ (Fig. 3c). Furthermore, an *in situ* electrochemical impedance spectroscopy technique was employed at various applied biases to track the interfacial charge-transfer ability during the OER process (Fig. 3d and e). Analysis of the Bode phase plots shows that the phase angles of RuO*_x_*/GDY decrease more rapidly than those of RuO*_x_* with increasing applied voltage, unveiling a faster interfacial charge transfer (Fig. 3f, Fig. S13 and Table S2). The electrochemically active surface area (ECSA) of RuO*_x_*/GDY was calculated to be 392 mF cm^−2^, which is 18.6 and 165 times larger than that of RuO*_x_* (21 mF cm^−2^) and GDY (2.4 mF cm^−2^), respectively (Fig. S14). The normalized OER polarization curves by the ECSA value implied that RuO*_x_*/GDY has a higher intrinsic activity (0.645 mA cm^−2^) than RuO*_x_* (0.149 mA cm^−2^) (Fig. 3g and Fig. S15). Besides, RuO*_x_*/GDY exhibits a higher exchange current density (*j*_0_) of 15.74 mA cm^−2^ than RuO*_x_* (7.76 mA cm^−2^) (Fig. 3g). Next, we investigated the OER activities of other samples that were subjected to different annealing temperatures. As shown in Fig. S16 and Table S3, RuO*_x_*/GDY displays the highest catalytic activity among these control samples. These results demonstrated that RuO*_x_*/GDY possesses both high catalytic activity and intrinsic activity.

Stability is another vital indicator of catalyst performance. As illustrated in Fig. S17, the activity of RuO*_x_*/GDY shows no significant degradation at 10, 100, 200 and 400 mA cm^−2^ after 5000 cycles of CV testing. Chronopotentiometry testing displays that the overpotential of RuO*_x_*/GDY increased by only 57 mV for 100 h of operation time, with a degradation rate of 0.57 mV h^−1^. As a comparison, the RuO*_x_* catalyst loses all activity after running for only 10 h (Fig. 3h). In terms of both activity and stability, RuO*_x_*/GDY outperforms most previously reported Ru-based catalysts (Fig. 3i and Table S4). The morphology and electronic structure of the catalyst obtained after the OER test were characterized by using XPS and TEM techniques (Fig. S18). The C 1*s* + Ru 3*d* XPS spectra revealed that the binding energy of the *sp*-C bond in GDY hardly shifted and only the Ru^0^ peak slightly shifted to a higher binding energy (∼0.1 eV) due to the inevitable oxidation of catalysts (Fig. S18a). There was almost no changes in the O 1*s* spectra (Fig. S18b and c). Moreover, HRTEM images (Fig. S18d and e) confirmed that GDY and Ru atom defects still existed in the spent RuO*_x_*/GDY catalyst. The corresponding EDS spectra (Fig. S18f) demonstrated a distribution of C, O and Ru elements throughout the sample. These results confirmed the excellent stability of RuO*_x_*/GDY during the acidic OER process.

**Figure 4.**
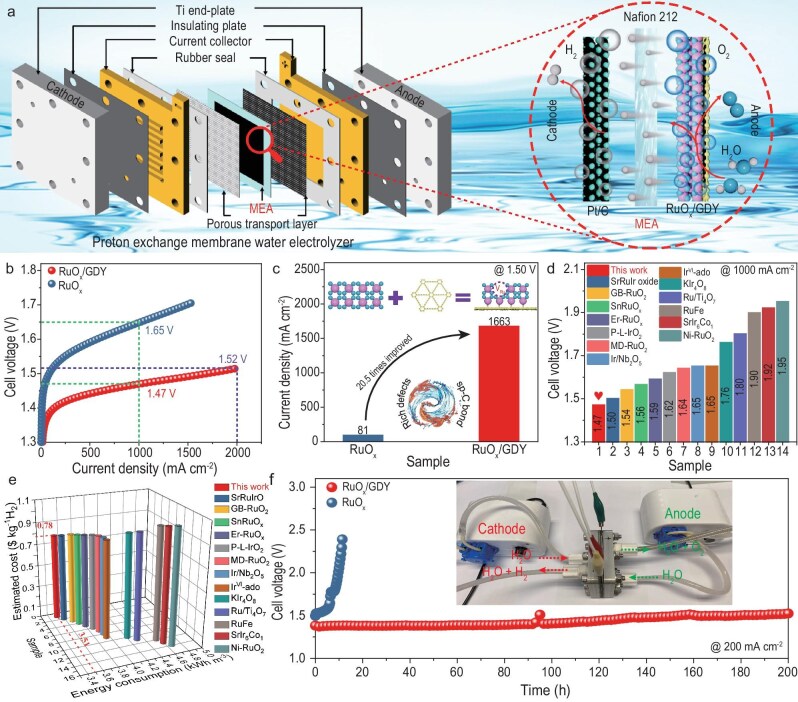
PEMWE device performance. (a) Structural model of the PEMWE set-up and reaction mechanism of water splitting in the membrane electrode assembly. (b) Polarization curves for RuO*_x_*/GDY and RuO*_x_* anode-based electrolysers. (c) Comparison of current density for RuO*_x_*/GDY and RuO*_x_* at a cell voltage of 1.50 V. (d and e) Comparison of cell voltage, device efficiency, energy consumption and estimated cost of RuO*_x_*/GDY with various catalysts at 1000 mA cm^−2^. (f) Stability curves of RuO*_x_*/GDY||Pt/C and RuO*_x_*||Pt/C at 200 mA cm^−2^ (inset: optical image of the PEMWE set-up).

To further investigate the practical applicability of RuO*_x_*/GDY catalysts, we integrated RuO*_x_*/GDY into a PEMWE system to assess its catalytic efficiency (Fig. 4a). As shown in Fig. 4b, the PEMWE set-up with a RuO*_x_*/GDY catalyst reached a current density of 1000 mA cm^−2^ at a cell voltage of 1.47 V, which is superior to that of RuO*_x_* (1.65 V). Besides, RuO*_x_*/GDY achieved a current density of 1663 mA cm^−2^ at 1.50 V, which is 20.5 times higher than that of RuO*_x_* (81 mA cm^−2^) (Fig. 4c). Further, the efficiency of the PEMWE set-up when using the RuO*_x_*/GDY catalyst reached 85.2% at 1000 mA cm^−2^. The corresponding energy consumption was 3.51 kWh Nm^−3^ H_2_, which is lower than those of the commercial PEMWE (∼5.0 kWh m^−3^ H_2_) and most reported catalysts (Fig. 4d and e) [43]. According to the international general calculation rules, the estimated cost per kg H_2_ produced is ∼US$0.78, which is significantly lower than the Department of Energy target of US$2.0 (Fig. 4e and Table S5) [12,44]. In addition, the PEMWE set-up with the RuO*_x_*/GDY catalyst can operate at 200 mA cm^−2^ for 200 h, which far exceeds RuO*_x_* (∼10 h) (Fig. 4f). These results demonstrated that GDY significantly enhances the performance of RuO*_x_* catalysts, showing promising prospects for practical applications in PEMWE set-ups.

### Insights into the origin of the acidic OER performances

**Figure 5.**
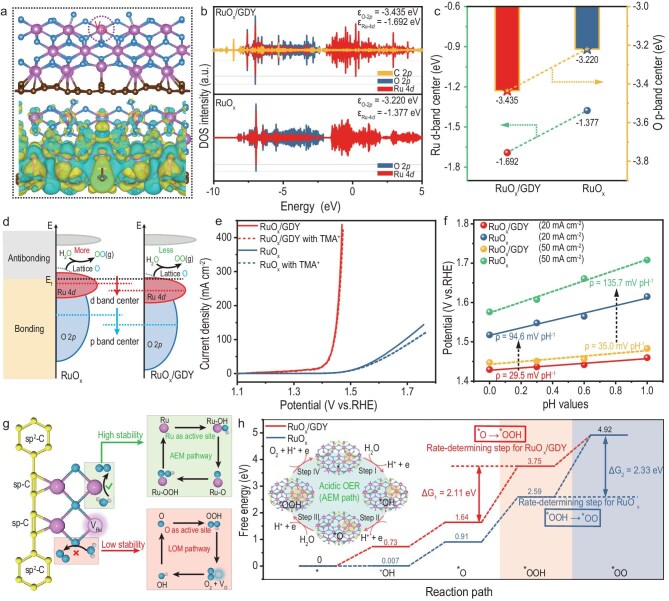
Understanding of the mechanism. (a) Structural model (top) and differential charge density (bottom) of RuO*_x_*/GDY with Ru atom defects. (b) Density of states plots for RuO*_x_*/GDY and RuO*_x_*. (c) Comparison of the Ru 4*d*-band centers and O 2*p*-band centers in RuO*_x_*/GDY and RuO*_x_*. (d) Schematic diagram of the band structures of RuO*_x_*/GDY and RuO*_x_*. (e) OER polarization curves for RuO*_x_*/GDY and RuO*_x_* with and without the addition of 0.05 M TMA^+^ cations in 0.5 M H_2_SO_4_. (f) pH dependences of the OER potential at 20 and 50 mA cm^−2^ for RuO*_x_*/GDY and RuO*_x_*. (g) Mechanistic scheme of the OER on RuO*_x_*/GDY. (h) Gibbs free energy diagrams of the OER on RuO*_x_*/GDY and RuO*_x_*.

For clarifying the underlying reasons for the improved OER performance of the RuO*_x_*/GDY catalyst, DFT calculations and some additional experiments were conducted. The structural models of RuO*_x_*/GDY with Ru atom defects and RuO*_x_* were constructed and optimized (Fig. 5a and Fig. S19). The charge density difference mapping (Fig. 5a and Fig. S20) highlights the obvious charge transfer from the C atoms in GDY to adjacent Ru atoms. The density of states (Fig. 5b and c) shows that the O-2*p* band center downshifts from −3.220 eV for RuO*_x_* to −3.435 eV for RuO*_x_*/GDY. This result implies that the lattice oxygen in RuO*_x_*/GDY is inactive and difficult to participate in the OER process (Fig. 5d). To confirm this conclusion, *in situ* probing reactions by introducing tetramethylammonium (TMA^+^) cations and pH-dependent experiments were performed. As shown in Fig. 5e, RuO*_x_*/GDY displays comparable OER activity regardless of the presence of the TMA^+^ cations; however, that of RuO*_x_* significantly decreases with TMA^+^ cations. In addition, compared with RuO*_x_*, the OER activity of RuO*_x_*/GDY exhibits a very weak dependence on the pH value, with slopes of 29.5 and 35.0 mV pH^−1^ at 20 and 50 mA cm^−2^, respectively (Fig. 5f and Fig. S21), which is indicative of the switch from the LOM to the classical adsorbate evolution mechanism (AEM) when GDY is integrated with RuO*_x_*. It is well established that the AEM pathway with metal atoms as redox centers can suppress the lattice oxygen participation in the OER reaction (LOM pathway), thereby alleviating catalyst dissolution and enhancing stability (Fig. 5g) [37].

The calculated 4*d*-band center of Ru in RuO*_x_*/GDY (−1.692 eV) is lower than that of RuO*_x_* (−1.377 eV) (Fig. 5b and c). In accordance with frontier orbital theory, this change means that the adsorption ability of oxygen intermediates on RuO*_x_*/GDY will decrease, helping to enhance electrocatalytic activity [45]. The Gibbs free energy profiles also demonstrated that the adsorption energies of the *OH, *O, *OOH and *OO intermediates on RuO*_x_*/GDY were lower than that of RuO*_x_* (Fig. 5h and Fig. S22). More importantly, the energy barrier in the rate-determining step is 2.11 eV for RuO*_x_*/GDY and the corresponding limiting overpotential is 0.88 V, which is lower than those of RuO*_x_* (2.33 eV and 1.10 V) (Fig. 5h and Fig. S23). These results confirmed that the coupling of GDY and RuO*_x_* enhances the intrinsic activity of RuO*_x_*.

## CONCLUSION

In summary, a RuO*_x_*/GDY catalyst with high-density Ru atom interface defects has been successfully constructed for acidic water oxidation via a sequential hydrolysis and pyrolysis method. As anticipated, the as-prepared RuO*_x_*/GDY catalyst exhibits superior OER performance, with overpotentials of 157 and 201 mV at 10 and 100 mA cm^−2^, together with wonderful catalytic stability. Especially when RuO*_x_*/GDY is used as an anode catalyst assembled in a PEMWE set-up, it also presents high catalytic activity (1.47 V at 1000 mA cm^−2^) and a low hydrogen production cost (US$0.78 per kg H_2_). Experimental and calculation results demonstrate that the robust *sp*-C–Ru bond that is formed between GDY and Ru atoms plays a key role in creating a high-density Ru atom defects interface and improving the activity and stability of RuO*_x_*. This work presents an effective strategy for the rational design of metal oxide electrocatalysts with high-density metal atom defects on the interface toward catalytic conversion.

## METHODS

### Materials

Ruthenium chloride hydrate (RuCl_3_•*x*H_2_O, 37.5 wt% Ru), CC, ammonium hydroxide (NH_3_•H_2_O, 30 wt%), copper foil (Cu, 0.1 mm thick), hydrochloric acid (HCl), nitric acid (HNO_3_), sulfuric acid (H_2_SO_4_, 1.84 g mL^−1^), acetone, dichloromethane (CH_2_Cl_2_) and pyridine were purchased from Sinopharm Chemical Reagent Co., Ltd. The deionized (DI) water that was used in all experiments was obtained from a Millipore system. Before the experiment, the CC was boiled in concentrated nitric acid (8 mol L^−1^) at 100°C for 48 h and then cleaned with deionized water and acetone.

### Preparation of porous GDY nanosheet

The GDY nanosheet was prepared by using the Glaser–Hay coupling method. Typically, the treated Cu foil (4.5 cm × 4.5 cm, two pieces) and treated CC (3.0 cm × 2.0 cm, two pieces) were placed in a glass reactor. Then, 20 mL of CH_2_Cl_2_ and 2 mL of pyridine solution containing 20 mg of hexaethynylbenzene were added into the above glass reactor and kept for 24 h at room temperature. After the reaction, the CC was removed and cleaned several times with acetone, deionized water and 1 M HCl solution, followed by drying in a vacuum oven at 60°C for 12 h, and the GDY nanosheet was finally obtained.

### Preparation of RuO*_x_*/GDY electrode

The RuO*_x_*/GDY electrode was prepared by using a three-step process involving adsorption, hydrolysis and pyrolysis. Firstly, 1.6 mL of RuCl_3_•*x*H_2_O (5 mg mL^−1^) solution was uniformly dripped onto a piece of GDY and kept at 50°C until the water had evaporated. Next, an excess NH_3_•H_2_O solution was added into the above GDY with RuCl_3_ to ensure complete hydrolysis of the Ru^3+^ ions (denoted as Ru(OH)*_x_*/GDY). The Ru(OH)*_x_*/GDY was then placed in a tube furnace and maintained at 300°C under Ar for 2 h. After the furnace had cooled down to room temperature, the obtained samples were sonicated for 5 s three times in a mixture of 10 mL of DI water and 10 mL of ethanol and dried in an oven at 80°C for 12 h.

### Preparation of RuO*_x_* electrode

The same procedure as for the RuO*_x_*/GDY electrode was used to prepare the RuO*_x_* electrode, except that the GDY was replaced by the bare CC.

## Supplementary Material

nwaf177_Supplemental_File
