# Supplementary material for: Controlled synthesis of high-density metal atom interface defects for acid water oxidation
Source: Natl Sci Rev. 2025 May 2;12(7):nwaf177. doi: 10.1093/nsr/nwaf177 (PMC12189214; doi:10.1093/nsr/nwaf177)
Supplement: nwaf177_Supplemental_File [file nwaf177_supplemental_file.pdf]

Supporting information

Controlled synthesis of high-density metal atom interface defects for acid water oxidation

Xinyu Ping[1], Yurui Xue,[1,2*] Han Wu[1,3], Siao Chen[1,3], Siyi Chen[1,3], Yang Gao[1], and Yuliang Li[1,3*]

[1]CAS Key Laboratory of Organic Solids, Institute of Chemistry, Chinese Academy of Sciences, Beijing, 100190 (P. R. China);

[2]State Key Laboratory of Supramolecular Structure and Materials, College of Chemistry, Jilin University, Changchun 130012 (P. R. China)

[3]University of Chinese Academy of Sciences, Beijing, 100190 (P. R. China)

E-mails: yrxue@jlu.edu.cn; ylli@iccas.ac.cn

Material characterizations

The crystalline structure of the samples was examined using powder X-ray diffractometer (XRD) on a PANalytical X'pert equipped with Cu Kα radiation (λ = 1.542 Å) at room temperature. The morphologies of the samples were characterized by a field emission scanning electron microscope (SEM, Hitachi SU-8020), a transmission electron microscope (TEM) and high-resolution TEM (HRTEM, JEM-2100). The energy dispersive X-ray spectrum (EDS) was employed to identify the types and distribution of elements. The electronic structure and chemical state of the samples were investigated by X-ray photoelectron spectroscopy (XPS) on Thermo Scientific ESCALab 250Xi instrument with Al Kα light source. Raman signals were recorded by Renishaw-2000 Raman spectrometer with 473 nm excitation laser. AFM images were obtained using a Bruker FASTSCANBIO at room temperature in non-contact mode. Electron paramagnetic resonance (EPR) spectra were collected on an EMXplus-9.5/12 with same sample loading. The K-edge X-ray absorption spectra (XAS) of Ru were recorded at the 1W1B beamline of the Beijing Synchrotron Radiation Facility in Beijing, China.

Electrochemical measurements

A conventional three-electrode system in a CHI760E electrochemical workstation was employed to evaluate the electrochemical performance of the samples. The prepared RuO_x/GDY and RuO_x electrode, Ag/AgCl (3.5 M KCl-saturated) and graphite rods (Φ = 6 mm) served as the working electrode (WE), reference electrode (RE) and counter electrode (CE), respectively, with a 0.5 M H_2SO_4 solution used as the electrolyte. All potentials were calibrated relative to the reversible hydrogen electrode (RHE) with iR compensation, according to the following calculations:

$$E_{RHE} = E_{Ag/AgCl} + 0.197\ V + 0.059 \times pH - I_{mea} \times R_{sol} \#(1)$$

where $E_{Ag/AgCl}$ is the potential relative to the Ag/AgCl electrode, which is the set potential during all measurements. I_{mea} is the measured polarization current. R_{sol} is the solution resistance.

To evaluate the OER activity, cyclic voltammetry (CV) was firstly employed to stabilize the catalyst between 1.0 and 1.6 V (vs. RHE) at a scan rate of 50 mV s^{-1} for 50 cycles in O_2-saturated 0.5 M H_2SO_4 solution. Subsequently, the polarization curves between 1.0 and 1.8 V (vs. RHE) were recorded via linear sweep voltammetry (LSV).

The electrochemical double-layer capacitance (C_{dl}) and electrochemically active surface area (ECSA) were measured. Specifically, the C_{dl} value was obtained by calculating the slope between the current density difference ($\Delta J = (J_a - J_b)/2$) and the scan rate (10 mV s^{-1}, 20 mV s^{-1}, 30 mV s^{-1}, 40 mV s^{-1} and 50 mV s^{-1}) at 1.1 V (vs. RHE, non-Faradaic area). The ECSA values were evaluated using the following formula: $ECSA = C_{dl}/C_s$, where C_s is ideally plating capacitance, with a value of 0.035 mF cm^{-2}.

Electrochemical impedance spectroscopy (EIS) was obtained in the frequency range from 10^6 Hz to 10^{-1} Hz at a voltage range of 1.2 -1.6 (V vs. RHE) with a 10 mV amplitude. The obtained EIS data was analyzed by Zview software to extract the key parameters, eg, solution resistance (R_{sol}) and charge transfer resistance (R_{ct}) and so on.

In our experiment, the stability of the catalysts was investigated by accelerated durability test (ADT) based on CVs and chronopotentiometry (CP). For ADT method, 50 cycles of CVs were conducted at potential windows from 1.0 to 1.6 (V vs. RHE) to motivate the catalyst, and measured its activity via recording LSV curves between 1.0 to 1.8 (V vs. RHE) as the initial state. Subsequently, LSV curves were also recorded after 500th, 1000th, 2000th, 3000th and 5000th CV cycles, respectively. For the CP test, a current density of 10 mA cm^{-2} was held and recorded the E-t curve.

PEMWE tests

The home-made PEMWE was assembled with RuO_x/GDY as anode catalysts (~0.5 mg_{Ru} cm^{-2}), commercial Pt/C (40 wt%, JM) as cathode catalyst (~0.4 mg_{Pt} cm^{-2}), Nafion 212 membrane as solid polymer electrolytes, Ti fiber and carbon paper as anode and cathode porous transport layer (PTL), respectively. To prepare the cathode catalyst ink, the Pt/C was dispersed into a mixture of Nafion (5 wt%), ethanol and DI. And then ultrasonicated for 30 min until a homogeneous catalyst ink was formed. The obtained cathode catalyst ink was sprayed onto one side of the PEM (1

cm ×1 cm reactive area). Subsequently, the PEM supported with catalyst, Ti fiber (on the anode side) and carbon paper (on the cathode side) were pressed together to construct the membrane electrode assembly (MEA). The PEMWE was operated at the temperature of 65 °C and inlet DI to the anode side as the electrolyte solution at a flow rate 30 mL min^{-1}. The performance of the PEMWE was evaluated by collecting polarization curves ranging from 0.8 V to 1.8 V. The stability was also evaluated by chronopotentiometry at 200 mA cm^{-2}.

The energy efficiency, energy consumption and hydrogen production cost of the PEMWE were calculated according to previously reported literature[1]. The specific descriptions are as follows:

Energy consumption calculation: The energy consumption of a PEMWE is calculated by the equation:

$$Energy\ consumption = \frac{U_{cell} \times I_{cell} \times t}{V_{H_2}} \#(2)$$

However, V_{H_2} can be calculated by Faraday's laws of electrolysis:

$$V_{H_2} = \frac{I_{cell} \times t \times 3600}{z \times F} \times V_m \#(3)$$

This formula can be simplified to:

$$Energy\ consumption = \frac{U_{cell} \times z \times F}{3600 \times V_m} \#(4)$$

Where U_{cell} is cell voltage (V) to deliver a current density of 1000 mA cm^{-2}; z is the number of electrons transferred to produce one hydrogen molecule, its value is 2; F is Faraday's constant (96484 C mol^{-1}); V_m is the molar volume of gas at 101.325 kPa and 273.15 K (0.0224 m^3 mol^{-1}).

Energy efficiency calculation: The energy efficiency of a PEMWE is calculated by the equation:

$$Energy\ efficiency = \frac{1.23\ V}{U_{cell}} \#(5)$$

where 1.23 V is the theoretical splitting voltage of water.

Hydrogen production cost: The electricity cost is calculated based on the energy consumption and electricity bill:

$$Hydrogen\ production\ cost = 0.2778 \times \frac{U_{cell} \times z \times F}{M_{H_2}} \times y \#(6)$$

where M_{H_2} is the relative molecular mass of hydrogen molecule (2 g mol^{-1}); y is electricity bill, which is obtained from the previous reports ($0.02 *per* kWh).

Theoretical calculations

All the density functional theory (DFT) calculations were conducted using the Vienna Ab initio Simulation Package (VASP). The interaction between ion cores and valence electrons was described by the generalized gradient approximation (GGA) of Perdew-Burke-Ernzerhof (PBE) and the projector augmented wave (PAW) method. The kinetic cutoff energy of the plane-wave for the one-electron wavefunction was set at 450 eV. The self-consistency convergence criterion for force and energy for each atom were 0.02 eV/Å and 10^{-6} eV, respectively. To eliminate periodic interference in the whole systems, a vacuum space with a height of 20 Å was fixed in the direction perpendicular to the surface of the structural model.

Based on structural characterization in the main text, a unit cell of pristine RuO_x model was built with the help of the standard RuO_2 model. The structural model of RuO_x/GDY was constructed, including a 2×2 supercell of graphdiyne (GDY) with a single layer (72 C atoms), a 2×5 supercell of RuO_2(200) with three layers (29 Ru atoms and 50 O atoms) and a Ru vacancy as the substrate. Reciprocal space was sampled using Monkhorst–Pack mesh k-space sampling grids with a 3×3×1 gamma k-point grid to optimize all the models. For the calculations of the density of states (DOS) of the sample, much denser 5×5×1 k-point grids were chosen.

To compare the intrinsic catalytic activity, models of three reaction intermediates (*OH, *O and *OOH) adsorbed onto the Ru site in RuO_x/GDY and RuO_x, based on the four elementary steps in the OER process, were constructed and all models were optimized to their most stable states. The free energy change (ΔG) was calculated using the computational hydrogen electrode model:

$$\Delta G = \Delta E_{ZPE} + \Delta E - T\times\Delta S + \Delta G_U \#(7)$$

where ΔE is the binding energy of the intermediates; ΔE_{ZPE} is the zero-point energy at 298.15 K; T is the experimental temperature (298.15 K); ΔS is the entropy change; ΔG_U is the free energy correction term for the electrode potential, which can be obtained by: $\Delta G_U = -eU$, in which U refers to the electrode potential with respect to

the standard hydrogen electrode, and e is the transferred charge.

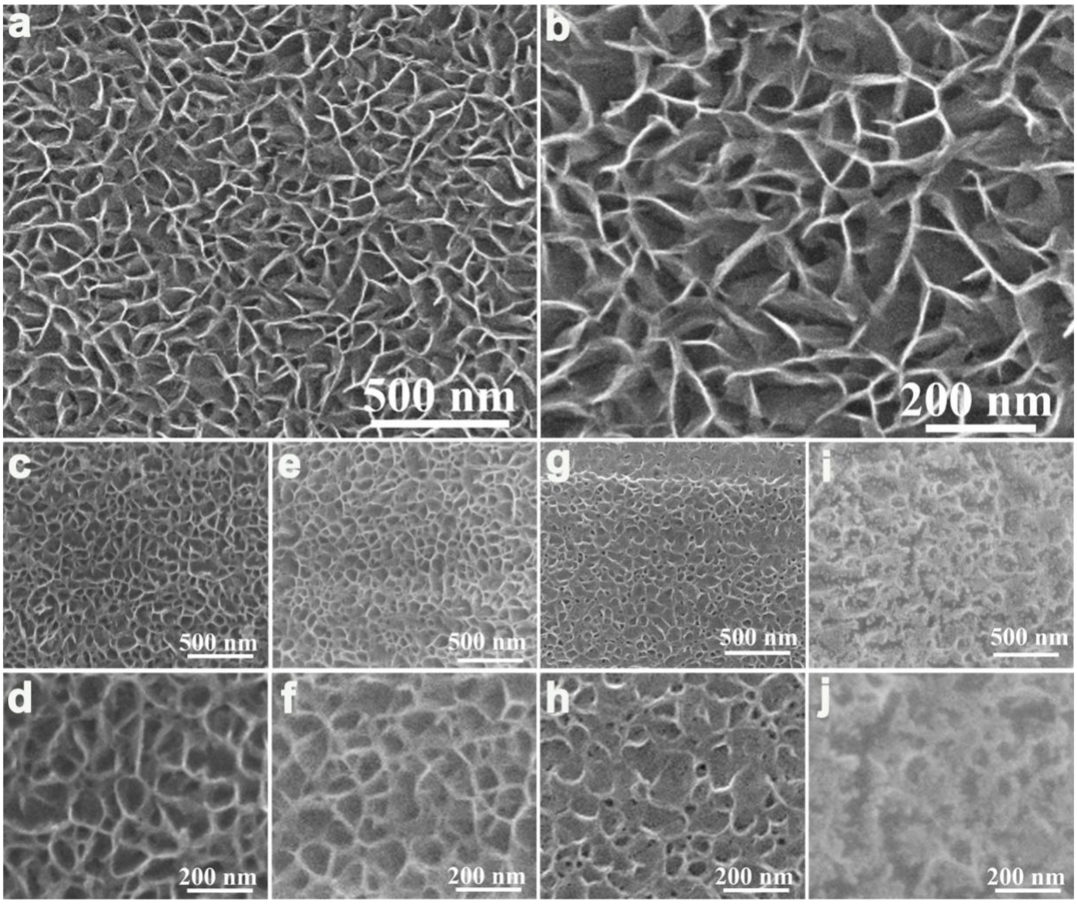

Fig. S1 SEM image of GDY (a-b) and RuO_x/GDY at 25 °C (c-d), 200 °C (e-f), 300 °C (g-h) and 400 °C (i-j).

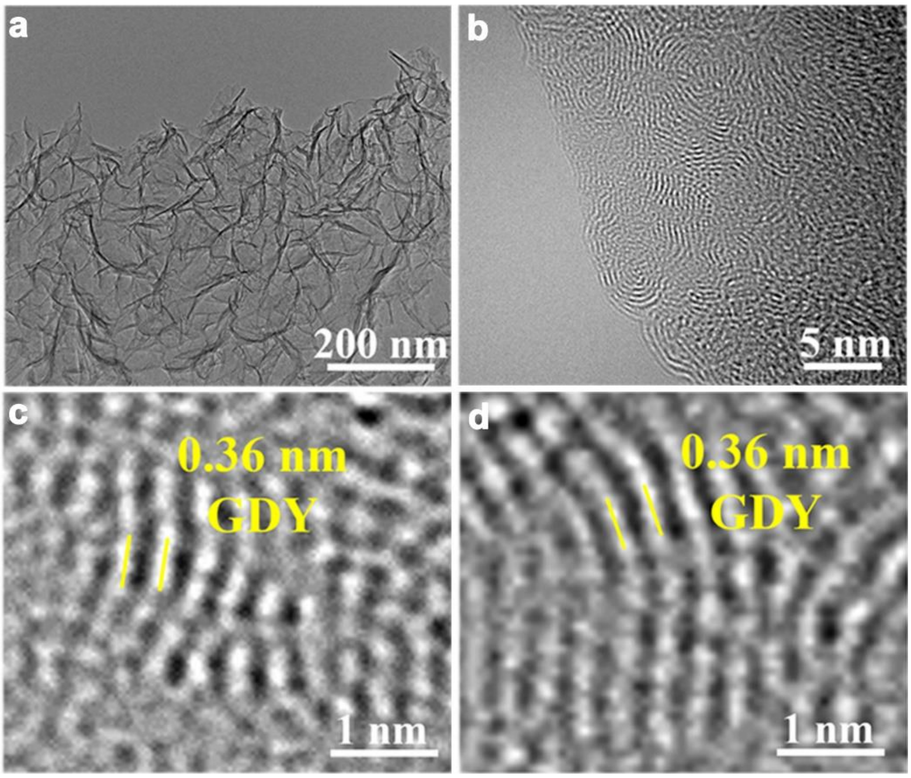

Fig. S2 TEM image of pure GDY.

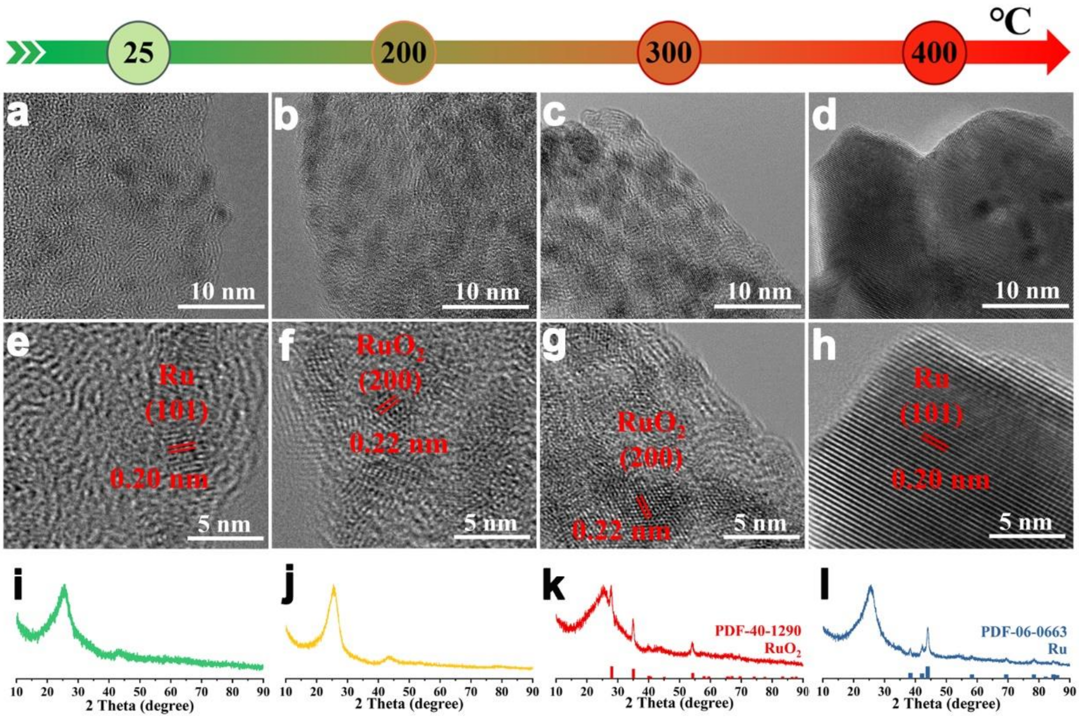

Fig. S3 (a-h) HRTEM images of the obtained RuO_x/GDY samples at different temperatures (25 °C, 200 °C, 300 °C and 400 °C); XRD patterns of RuO_x/GDY at 25 °C (i), 200 °C (j), 300 °C (k) and 400 °C (l).

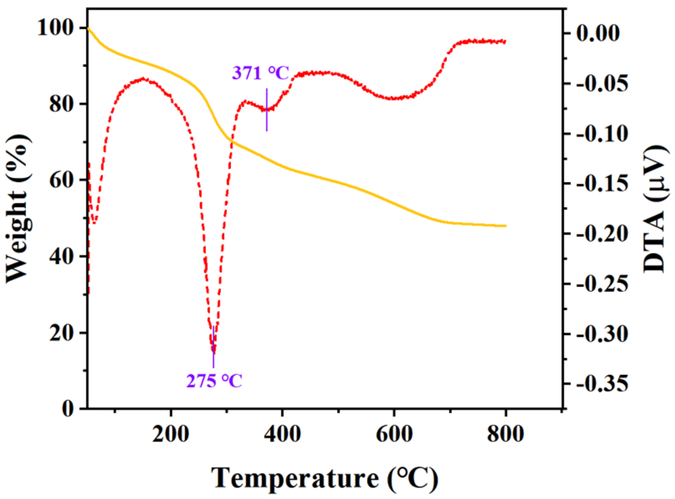

Fig. S4 TGA curve of $Ru(OH)_x$/GDY sample.

TGA curve shows two endothermic peaks at 275 °C and 371 °C, corresponding to the dehydration of Ru(OH)x to form RuOx and the conversion of RuOx to metallic Ru processes, respectively.

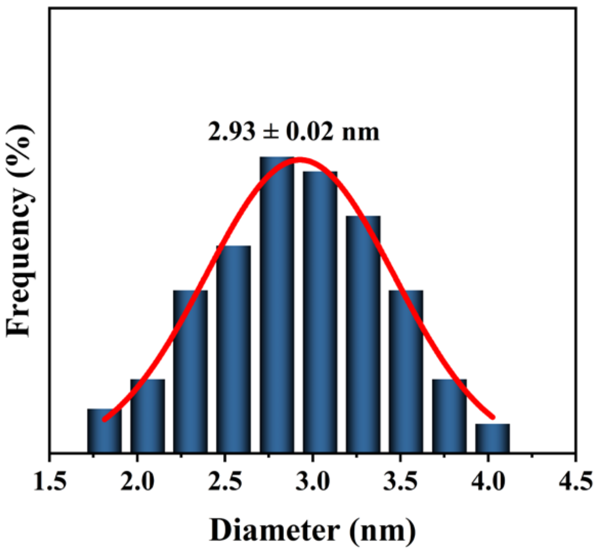

Fig. S5 Grain size distribution diagram of RuO_x/GDY.

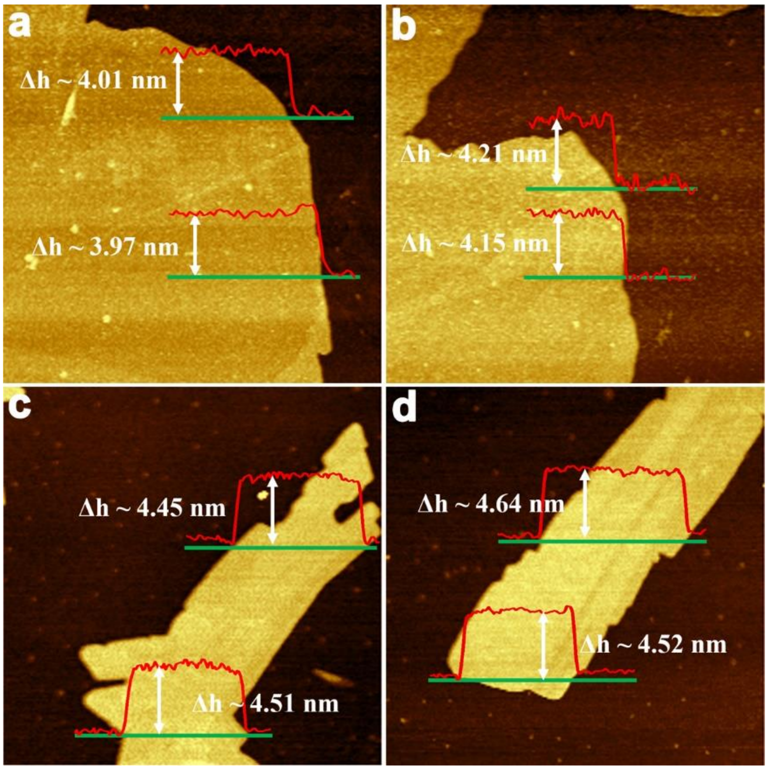

Fig. S6 AFM images of GDY (a-b) and (c-d) RuO_x/GDY

The average thickness of GDY is 4.09 nm, while the average thickness of RuO_x/GDY is 4.53 nm, so the average thickness of RuO_x on loaded GDY is 0.44 nm.

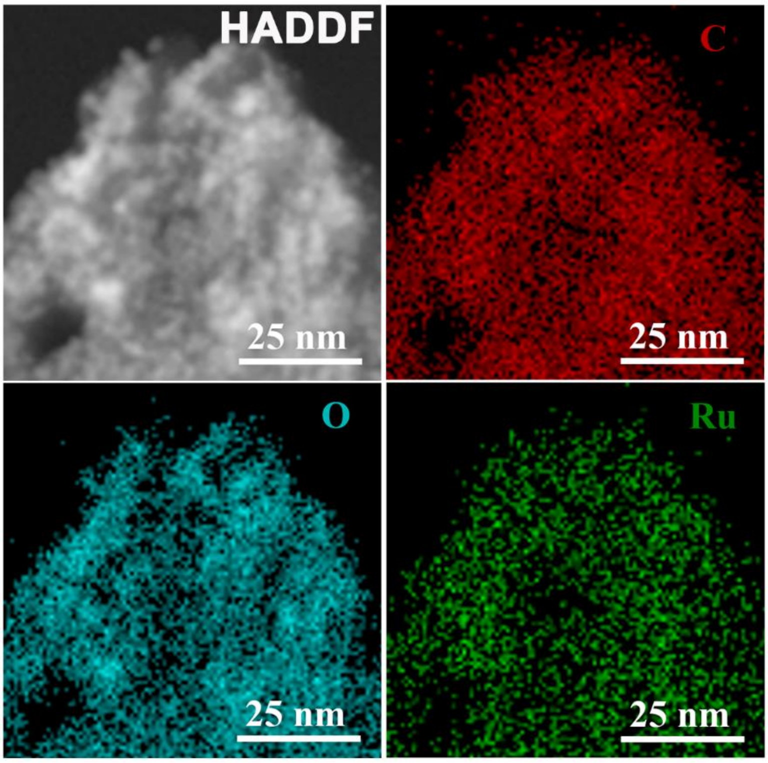

Fig. S7 EDS images of RuO_x/GDY

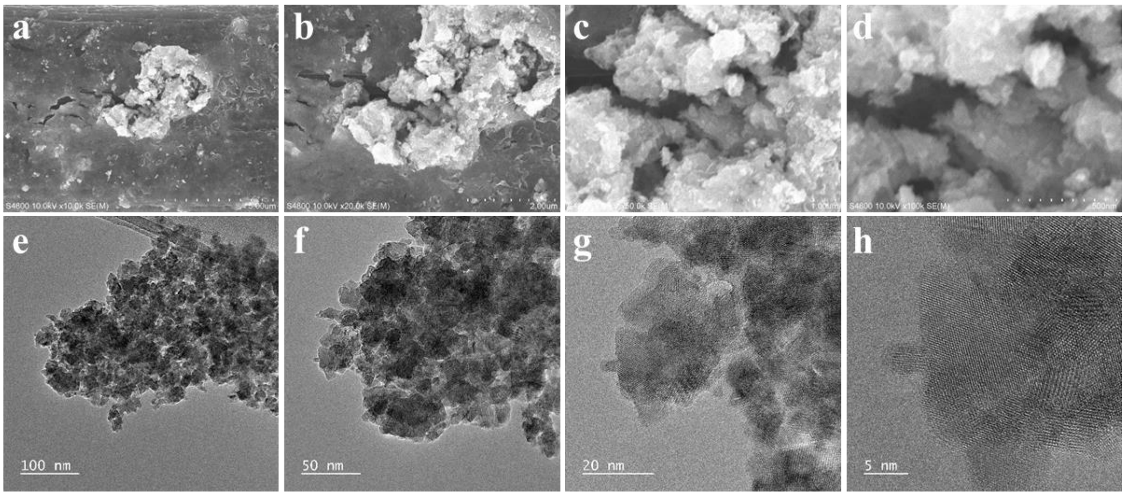

Fig. S8 (a-d) SEM images of RuO_x sample different scales; (e-h) TEM images of RuO_x sample different scales.

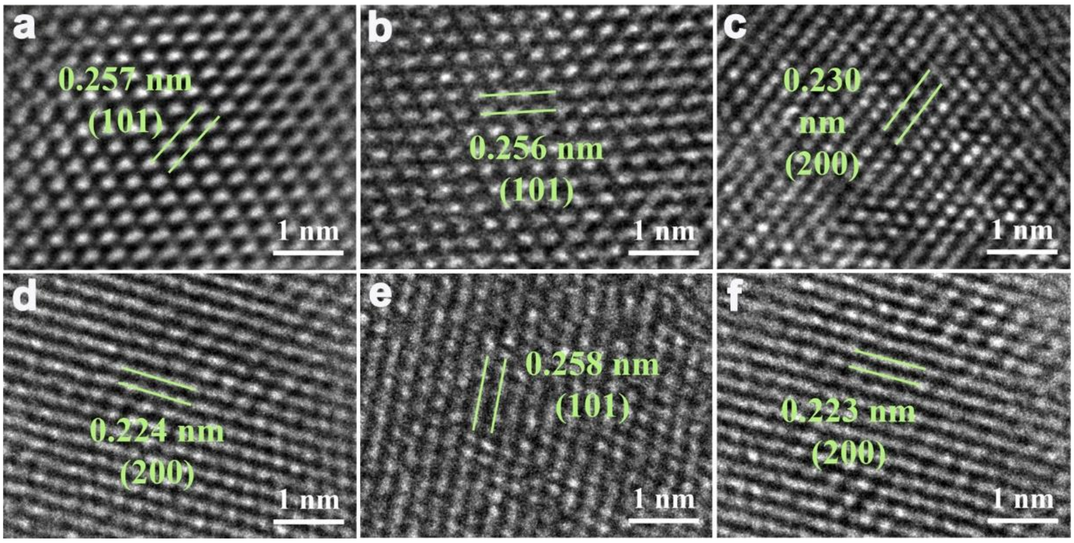

Fig. S9 HRTEM images of RuO_x.

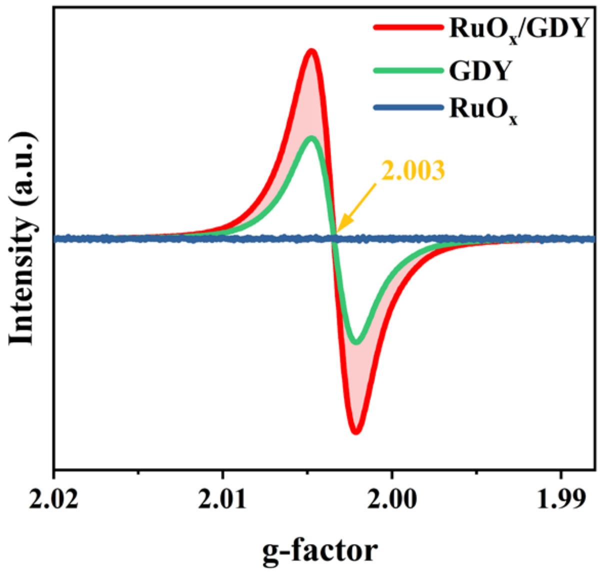

Fig S10. EPR spectra of RuO_x/GDY, RuO_x and GDY.

Although GDY exhibits a peak at g = 2.003, this peak is more pronounced for RuO_x/GDY at the same mass of the test sample, indicating that RuO_x/GDY has rich Ru vacancies.

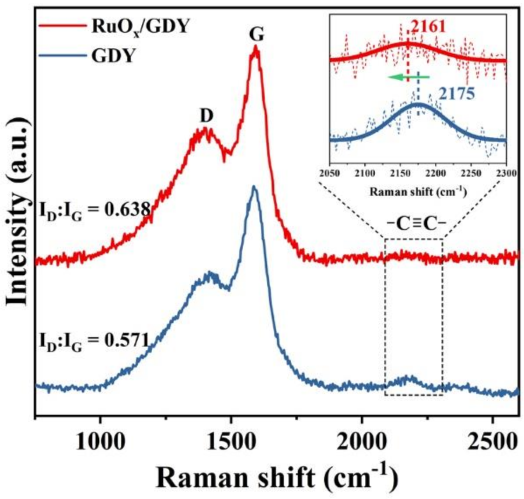

Fig. S11 Raman spectroscopy of RuO_x/GDY and GDY.

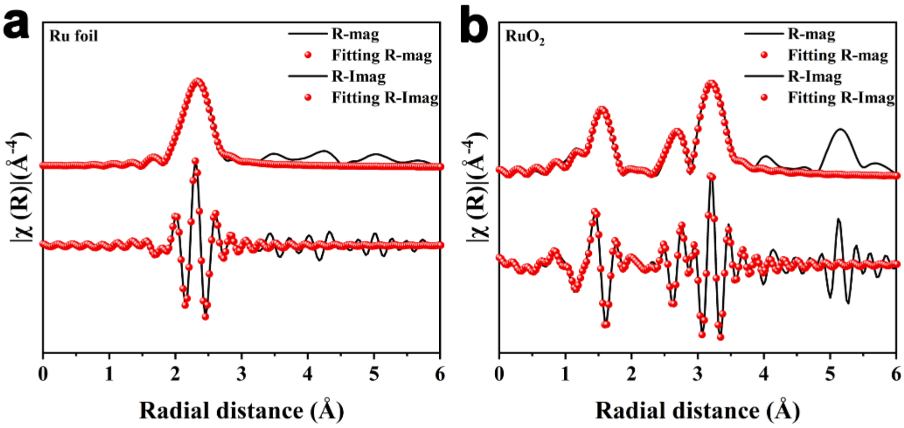

Fig. S12 EXAFS fitting in R-space of (a) Ru foil and (b) RuO_2. R-mag represents the magnitude of the EXAFS data in R-space; R-Imag is the imaginary part of the EXAFS data in R-space.

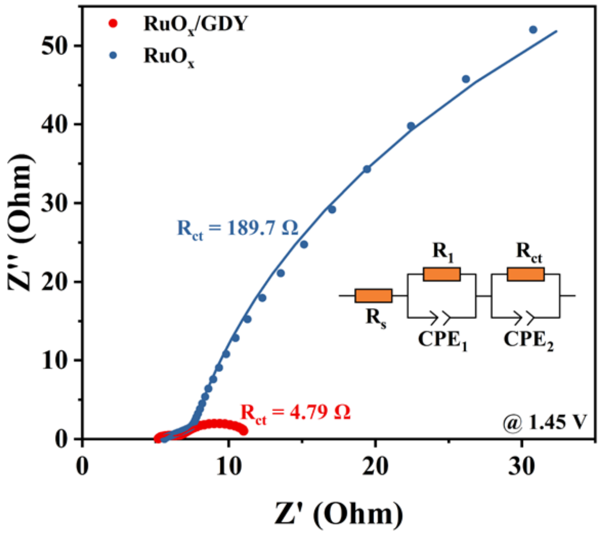

Fig. S13 EIS plots of RuO_x/GDY (d) and RuO_x (e) at 1.45V (vs. RHE) (inset: The fitting model of EIS plots).

Specifically, the EIS plots of RuO_x/GDY and RuO_x at 1.4 V were fitted. The fitting results show that the charge transfer resistance (R_{ct}) of RuO_x/GDY is 4.79 Ω, far smaller than that of RuO_x (189.7 Ω) (Table S2).

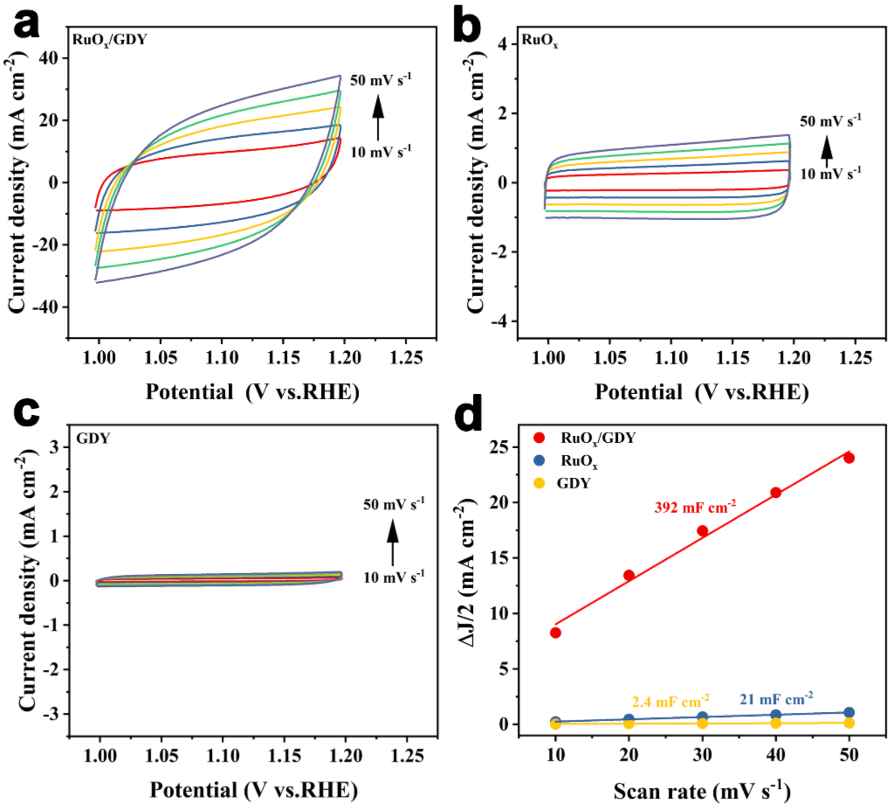

Fig.S14 Measurement of the double layer capacitance of (a) RuO_x/GDY, (b) RuO_x, (c) GDY; (d) the calculated ECSA inferred from CV curves.

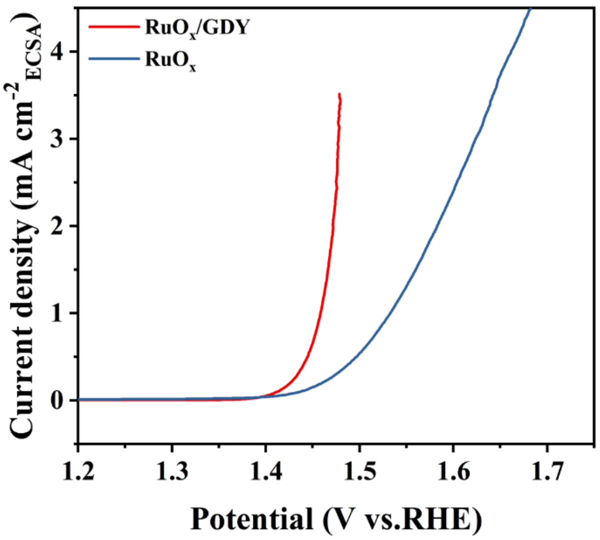

Fig. S15 Polarization curves for RuO_x/GDY and the RuO_x normalized by ECSA value.

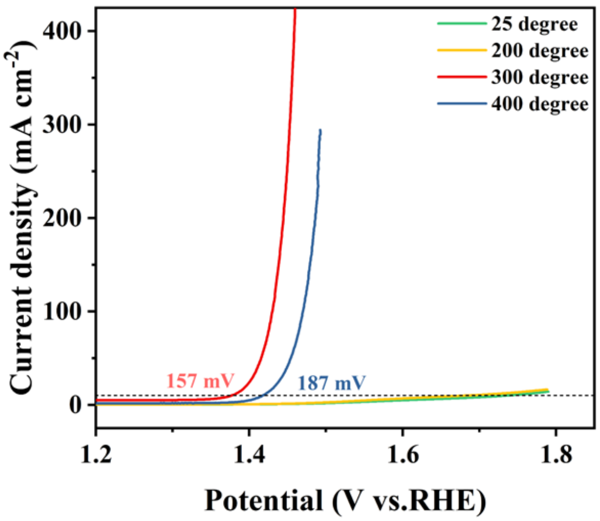

Fig.S16 Polarization curves for RuO_x/GDY under different annealing temperature.

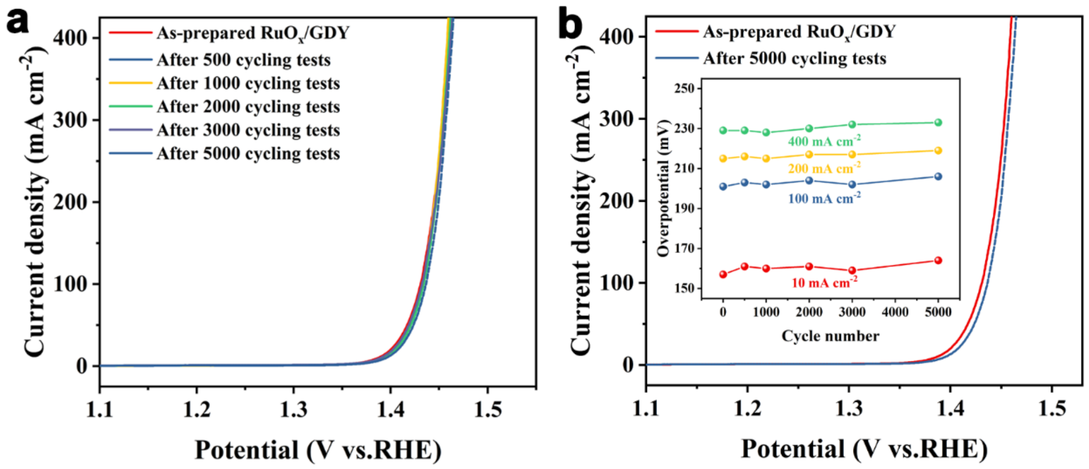

Fig. S17 (a) Polarization curves for RuO_x/GDY under different CV cycling tests (500 CVs, 1000 CVs, 2000 CVs, 3000 CVs and 5000 CVs). (b) LSV curves of RuO_x/GDY before and after 5000 CVs test and the change of overpotential at 10 mA cm^{-2}, 100 mA cm^{-2}, 200 mA cm^{-2} and 400 mA cm^{-2}.

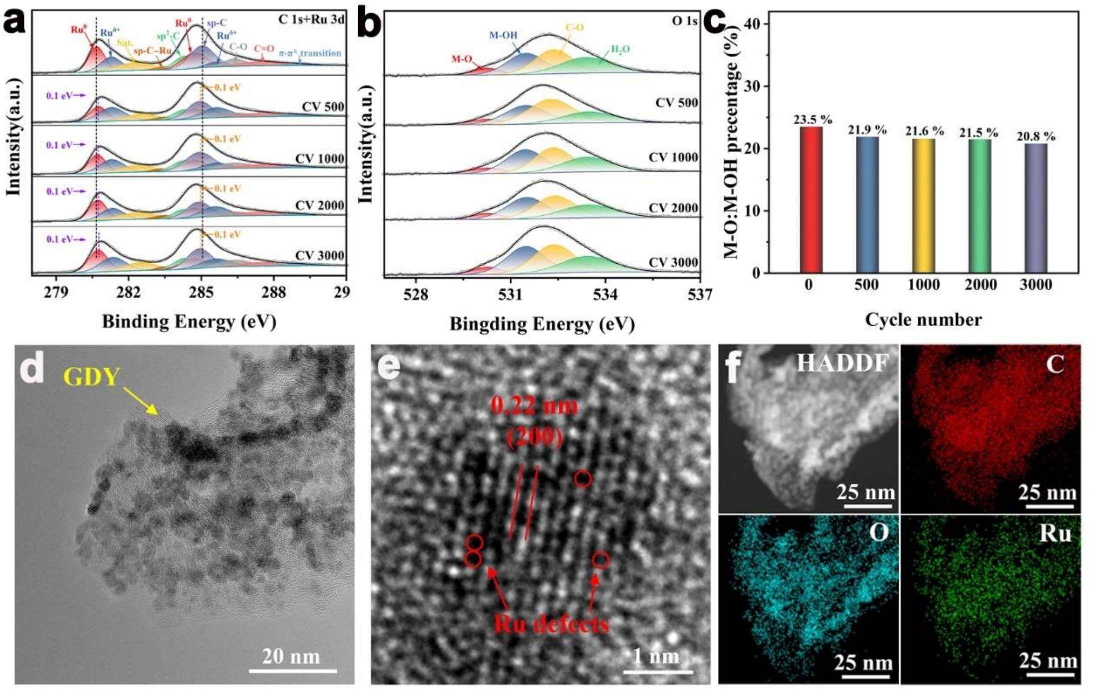

Fig. S18 (a) C1s and Ru 3d XPS spectra; (b) O1s XPS spectra; (c) the ratio of M-O and M-OH; (d) TEM, (e) HETRM and (f) Corresponding EDS spectrum of RuO_x/GDY after 5000 CVs test.

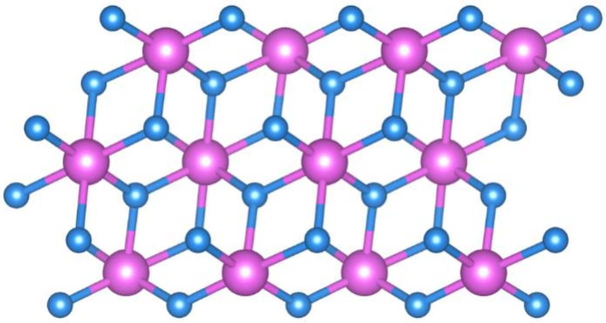

Fig. S19 The structural model of RuO_x.

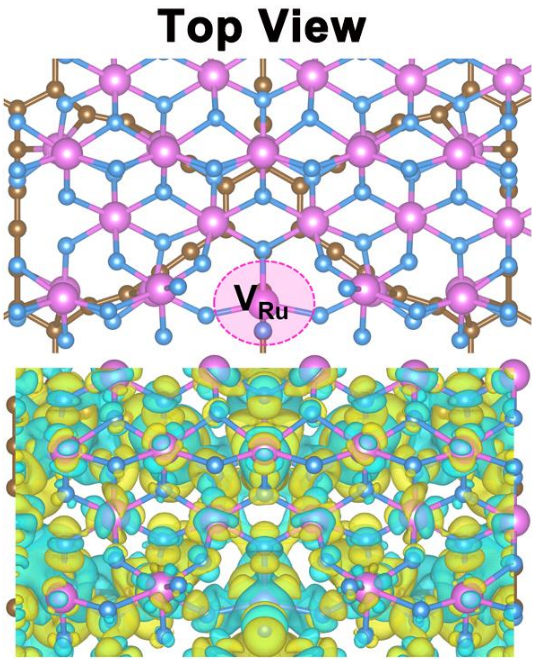

Fig. S20 The structural model (top) and differential charge density (bottom) of RuOx/GDY with Ru atom defects (Top view).

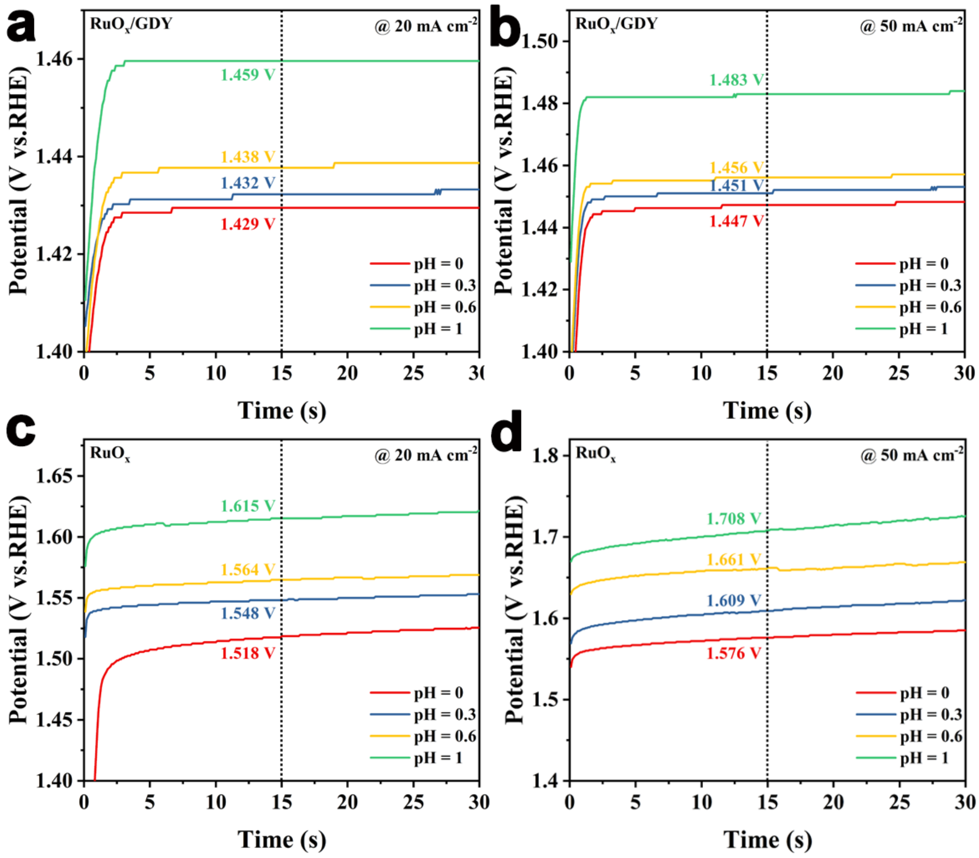

Fig. S21 CP curves of RuO_x/GDY at different pH values under 20 mA cm^{-2} (a) and 50 mA cm^{-2} (b) conditions; CP curves of RuO_x at different pH values under 20 mA cm^{-2} (c) and 50 mA cm^{-2} (d) conditions.

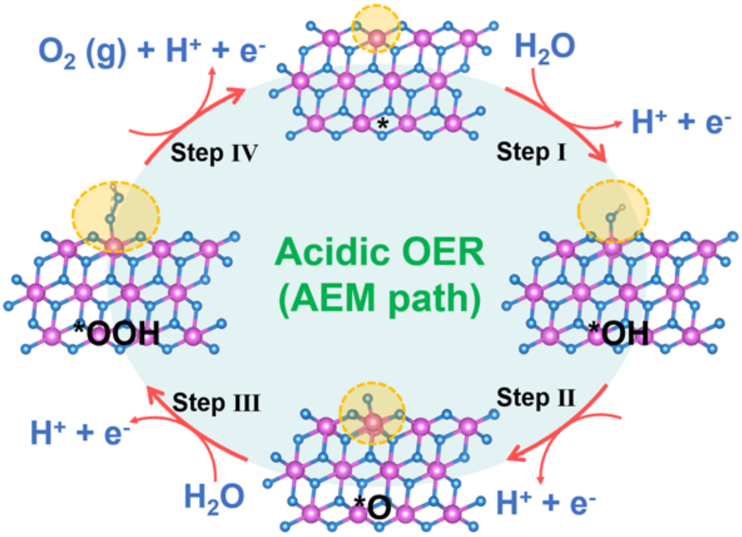

Fig. S22 Typical OER process with the four-electron reaction pathway for RuO_x, the yellow area represents the location of the active site.

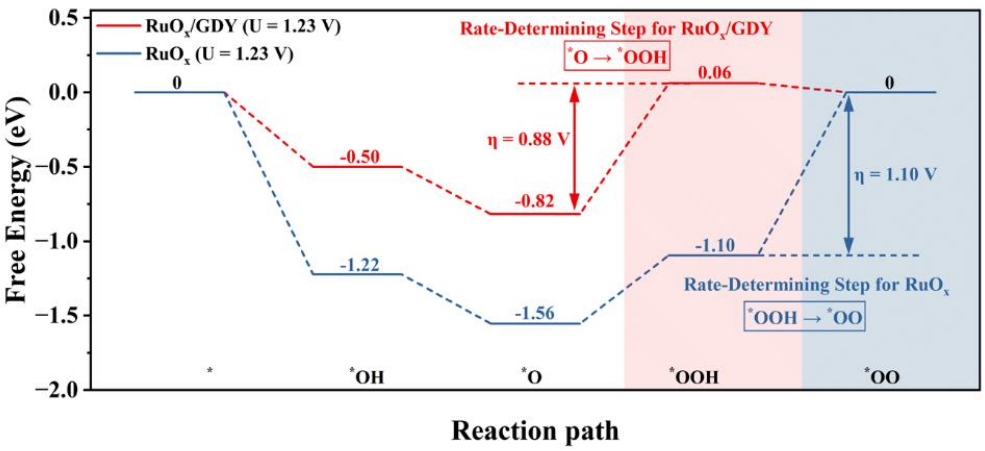

Fig. S23 Gibbs free energy diagrams of the OER on RuO_x/GDY and RuO_x at U = 1.23 V.

Table S1 EXAFS fitting parameters at the Ru K-edge for various samples ($S0^2$=0.96)

	shell	CN	R(Å)	σ^2	E_0	ΔE_0	R factor
Ru foil	Ru-Ru	6	2.62±0.008	0.0003	22118	4.69±1.15	0.012
	Ru-Ru	6	2.71±0.009	0.0018		12.86±2.02	
RuO_2	Ru-O	6	1.97±0.004	0.0025	22135	4.16±2.15	0.017
	Ru-Ru	2	3.08±0.005	0.0025		16.35±4.78	
	Ru-Ru	8	3.55±0.004	0.0033		5.61±1.59	
RuO_x/GDY	Ru-O/C	4.07±0.27	2.01±0.01	0.0069	22126	0.06±0.85	0.014
	Ru-Ru	3.04±0.73	3.11±0.03	0.0064		7.65±1.64	
	Ru-Ru	2.29±1.53	3.37±0.06	0.0060		1.13±7.20	

CN: coordination numbers; R: bond distance; σ^2: Debye-Waller factors; ΔE_0: the inner potential correction. R factor: goodness of fit. $S0^2$ was set to 0.96, according to the experimental EXAFS fit of Ru foil reference by fixing CN as the known crystallographic value.

Table S2 Results of EIS analysis at 1.4 V for RuO_x/GDY and the RuOx electrocatalysts

	**RuO_x/GDY**	**RuO_x**
**R_s**	5.12	5.64
**R_1**	1.66	1.91
**CPE_1-T**	0.0022	0.0026
**CPE_1-P**	0.649	0.688
**R_{ct}**	4.79	189.7
**CPE_2-T**	0.0119	0.0029
**CPE_2-P**	0.870	0.910

Table S3 The overpotential of RuO_x/GDY at 10 mA cm^{-2} and 100 mA cm^{-2} under different annealing temperature.

	**25 ℃**	**200 ℃**	**300 ℃**	**400 ℃**
**10 mA cm^{-2}**	450	511	157	187
**100 mA cm^{-2}**			201	240

Table S4 Comparison of the OER performance with recently reported Ru-based oxide electrocatalysts in acidic media.

**Catalyst**	**Overpotential (mV@10 mA cm^{-2})**	**Tafel slop**	**Stability (h) @ 10 mAcm^{-2}**	**Degradation rate (μV h^{-1})**	**reference**
RuO_x/GDY	157	46.8	100	570	This work
$Li_{0.52}RuO_2$	156	83.5	70	1685	[2]
a/c RuO_2	205	48.6	60	1450	[3]
$Co_{0.11}Ru_{0.89}O_2$	169	49	50	1580	[4]
C-RuO_2-RuSe-10	242	50.4	50	3840	[5]
Mg-RuO_2	228	48.66	30	4666	[6]
$CaCu_3Ru_4O_{12}$	171	40	24	875	[7]
75-H-RuO_2	200	71	20	3950	[8]
Ufd-RuO_2/CC	179	36.9	20	950	[9]
$Mn_{0.73}Ru_{0.27}O_2$	208	65.3	10	5200	[10]
Mn-RuO_2	158	42.94	10	19200	[11]
$Cr_{0.6}Ru_{0.4}O_2$	178	58	10	5600	[12]
Cu-doped RuO_2	188	43.96	8	10375	[13]
RuO_2 NSs	199	38.2	6.9	5940	[14]
YBRO-0.15	278	40.8	5	4583	[15]
$Y_{0.85}Mg_{0.15}Ru_2O_7$	258	39.1	5	1301	[16]

Table S5 Comparison of cell voltage, device efficiency, energy consumption and estimated cost of RuO_x/GDY with recently reported acidic OER catalysts at 1 A cm^{-2}.

**Catalyst**	**Cell voltage (V@1A cm^{-2})**	**Device efficiency (%)**	**Energy consumption (kWh m^{-3})**	**Estimated cost (US$ kg^{-1})**	**reference**
RuO_x/GDY	1.47	85.2	3.51	0.78	This work
SrRuIr oxide	1.5	83.5	3.59	0.80	[17]
GB-RuO_2	1.541	81.3	3.69	0.83	[18]
$SnRuO_x$	1.565	80.0	3.74	0.84	[19]
Er-RuO_x	1.59	78.8	3.80	0.85	[20]
p-L-IrO_2	1.62	77.3	3.87	0.87	[21]
MD-RuO_2-BN	1.64	76.4	3.92	0.88	[22]
Ir/Nb_2O_5	1.65	75.9	3.94	0.88	[23]
Ir^{VI}-ado	1.65	75.9	3.94	0.88	[24]
KIr_4O_8	1.68	74.5	4.02	0.90	[25]
$Ta_{0.1}Tm_{0.1}Ir_{0.8}O_{2-\delta}$	1.76	71.2	4.21	0.94	[26]
Ru/Ti_4O_7	1.8	69.6	4.31	0.96	[27]
RuFe	1.898	66.0	4.54	1.02	[28]
$SrIr_6Co_1$	1.92	65.2	4.59	1.03	[29]
Ni-RuO_2	1.95	64.3	4.66	1.05	[30]

Reference

1. Rong C, Wang S, Shen X et al. Defect-balanced active and stable Co_3O_{4-x} for proton exchange membrane water electrolysis at ampere-level current density. *Energy Environ Sci* 2024; **17**: 4196-4204.
2. Qin Y, Yu T, Deng S et al. RuO_2 electronic structure and lattice strain dual engineering for enhanced acidic oxygen evolution reaction performance. *Nat Commun* 2022; **13**: 3784.
3. Zhang L, Jang H, Liu H et al. Sodium-decorated amorphous/crystalline RuO_2 with rich oxygen vacancies: A robust pH-universal oxygen evolution electrocatalyst. *Angew Chem Int Ed* 2021; **60**: 18821-18829.
4. Tian Y, Wang S, Velasco E et al. A Co-doped nanorod-like RuO_2 electrocatalyst with abundant oxygen vacancies for acidic water oxidation. *iScience* 2020; **23**: 100756.
5. Wang J, Cheng C, Yuan Q et al. Exceptionally active and stable RuO_2 with interstitial carbon for water oxidation in acid. *Chem* 2022; **8**: 1673-1687.
6. Li Z, Wang S, Tian Y et al. Mg-Doping improves the performance of Ru-based electrocatalysts for the acidic oxygen evolution reaction. *Chem Commun* 2020; **56**: 1749-1752.
7. Miao X, Zhang L, Wu L et al. Quadruple perovskite ruthenate as a highly efficient catalyst for acidic water oxidation. *Nat Commun* 2019; **10**: 3809.
8. He J, Chen W, Gao H et al. Tuning hydrogen binding modes within RuO_2 lattice by proton and electron co-doping for active and stable acidic oxygen evolution. *Chem Catalysis* 2022; **2**: 578-594.
9. Ge R, Li L, Su J et al. Ultrafine defective RuO_2 electrocatayst integrated on carbon cloth for robust water oxidation in acidic media. *Advanced Energy Materials* 2019; **9**: 1901313.
10. Wang K, Wang Y, Yang B et al. Highly active ruthenium sites stabilized by modulating electron-feeding for sustainable acidic oxygen-evolution electrocatalysis. *Energy Environ Sci* 2022; **15**: 2356-2365.
11. Chen S, Huang H, Jiang P et al. Mn-doped RuO_2 nanocrystals as highly active electrocatalysts for enhanced oxygen evolution in acidic media. *ACS Catal* 2020; **10**: 1152-1160.
12. Lin Y, Tian Z, Zhang L et al. Chromium-ruthenium oxide solid solution electrocatalyst for highly efficient oxygen evolution reaction in acidic media. *Nat Commun* 2019; **10**: 162.
13. Su J, Ge R, Jiang K et al. Assembling ultrasmall copper-doped ruthenium oxide nanocrystals into hollow porous polyhedra: highly robust electrocatalysts for oxygen evolution in acidic media. *Adv Mater* 2018; **30**: 1801351.
14. Zhao Z L, Wang Q, Huang X et al. Boosting the oxygen evolution reaction using defect-rich ultra-thin ruthenium oxide nanosheets in acidic media. *Energy Environ Sci* 2020; **13**: 5143-5151.
15. Feng Q, Zou J, Wang Y et al. Influence of surface oxygen vacancies and ruthenium valence state on the catalysis of pyrochlore oxides. *ACS Applied Materials & Interfaces* 2020; **12**: 4520-4530.
16. Feng Q, Zhang Z, Huang H et al. An effective strategy to tune the oxygen vacancy of pyrochlore oxides for electrochemical energy storage and conversion systems. *Chem Eng J* 2020; **395**: 124428.
17. Wen Y, Chen P, Wang L et al. Stabilizing highly active Ru sites by suppressing lattice oxygen participation in acidic water oxidation. *J Am Chem Soc* 2021; **143**: 6482-6490.
18. He W, Tan X, Guo Y et al. Grain-boundary-rich RuO_2 porous nanosheet for efficient and stable

acidic water oxidation. *Angew Chem Int Ed* 2024; **63**: e202405798.
19. Shi Z, Li J, Wang Y et al. Customized reaction route for ruthenium oxide towards stabilized water oxidation in high-performance PEM electrolyzers. *Nat Commun* 2023; **14**: 843.
20. Li L, Zhang G, Zhou C et al. Lanthanide-regulating Ru-O covalency optimizes acidic oxygen evolution electrocatalysis. *Nat Commun* 2024; **15**: 4974.
21. Xie Z, Liang X, Kang Z et al. High-porosity, layered iridium oxide as an efficient, durable anode catalyst for water splitting. *CCS Chemistry* 2024; **0**: 1-13.
22. Chen D, Yu R, Yu K et al. Bicontinuous RuO_2 nanoreactors for acidic water oxidation. *Nat Commun* 2024; **15**: 3928.
23. Shi Z, Li J, Jiang J et al. Enhanced acidic water oxidation by dynamic migration of oxygen species at the Ir/Nb_2O_5−catalyst/support interfaces. *Angew Chem Int Ed* 2022; **61**: e202212341.
24. Li A, Kong S, Adachi K et al. Atomically dispersed hexavalent iridium oxide from MnO_2 reduction for oxygen evolution catalysis. *Science* 2024; **384**: 666-670.
25. Li Z, Li X, Wang M et al. KIr_4O_8 nanowires with rich hydroxyl promote oxygen evolution reaction in proton exchange membrane water electrolyzer. *Adv Mater* 2024; **36**: 2402643.
26. Hao S, Sheng H, Liu M et al. Torsion strained iridium oxide for efficient acidic water oxidation in proton exchange membrane electrolyzers. *Nat Nanotechnol* 2021; **16**: 1371-1377.
27. Zhao S, Hung S-F, Deng L et al. Constructing regulable supports via non-stoichiometric engineering to stabilize ruthenium nanoparticles for enhanced pH-universal water splitting. *Nat Commun* 2024; **15**: 2728.
28. Chen J, Ma Y, Huang T et al. Ruthenium-based binary alloy with oxide nanosheath for highly efficient and stable oxygen evolution reaction in acidic media. *Adv Mater* 2024; **36**: 2312369.
29. Zhao J-W, Yue K, Zhang H et al. The formation of unsaturated IrO_x in $SrIrO_3$ by cobalt-doping for acidic oxygen evolution reaction. *Nat Commun* 2024; **15**: 2928.
30. Wu Z-Y, Chen F-Y, Li B et al. Non-iridium-based electrocatalyst for durable acidic oxygen evolution reaction in proton exchange membrane water electrolysis. *Nature Materials* 2023; **22**: 100-108.
